# Study on the value of small dense low‐density lipoprotein in predicting cardiovascular and cerebrovascular events in the high‐risk stroke population

**DOI:** 10.1002/jcla.24278

**Published:** 2022-03-02

**Authors:** Hanlu Shi, Jianwei Guo, Ke Xu, Fujie Zhang, Yonglie Zhou

**Affiliations:** ^1^ 70571 School of Medical Technology and Information Engineering Zhejiang Chinese Medical University Hangzhou China; ^2^ Laboratory Medicine Center Department of Clinical Laboratory Zhejiang Provincial People’ s Hospital, Affiliated People's Hospital, Hangzhou Medical College Hangzhou China; ^3^ Department of Clinical Laboratory The Second Hospital of Jiaxing Jiaxing China; ^4^ Qian Xi Nan Hospital of Traditional Chinese Medicine Qian Xi Nan Buyei and Miao Autonomous Prefecture China

**Keywords:** atherosclerosis, cardiovascular and cerebrovascular events, lipid, sdLDL, stroke

## Abstract

**Background:**

Lipid management in people at high risk of stroke is an important measurement to prevent the occurrence of stroke. The study aims to investigate the association between sdLDL and cardiovascular and cerebrovascular events in high‐risk stroke populations.

**Methods:**

This was a prospective study. Screened from 15,933 individuals aged >40 years in April 2013 and followed up at 3rd, 6th, 12th, and 24th months, 823 participants met the screening criteria and were investigated for clinical data and biochemical parameters.

**Results:**

A total of 286 subjects had varying degrees of carotid stenosis, and 18 subjects experienced cardiovascular and cerebrovascular events during the two‐year follow‐up period. There was no positive correlation between sdLDL and carotid stenosis. Carotid stenosis and extent of carotid stenosis involvement did not predict cardiovascular and cerebrovascular events in patients with high‐risk stroke, while sdLDL did. The sdLDL level in the events group was significantly higher than those in the no event group (*p* = 0.002). In the events group, the risk of events in the fourth quartile of sdLDL was 10.136 times higher than in the first quartile (HR = 10.136, 95% CI: 1.298–79.180, *p* = 0.027).

**Conclusions:**

sdLDL was positively correlated with the incidence of cardiovascular and cerebrovascular events, which can predict the occurrence of an event and provide a scientific basis for early prevention.

## Graphical abstract

(a) The difference of 7 indicators between the group without cardiovascular and cerebrovascular events and the group with cardiovascular and cerebrovascular events, **p < 0.01. (b) Predictive values of serum lipids for cardiovascular and cerebrovascular events. The ROC curves and AUCs regarding risk are depicted in Figure 5. AUCs: 0.716 for sdLDL, 0.574 for TC, 0.583 for LDL, 0.576 for nonHDL, 0.538 for TG, 0.507 for HDL, 0.472 for Lpa. (c) sdLDL quartile grouping hazard function diagram. Q1: sdLDL ≤ 0.659 mmol/L; Q2: 0.659 mmol/L< sdLDL <0.926 mmol/L; Q3: 0.926 mmol/L≤ sdLDL <1.235 mmol/L; Q4: sdLDL ≥ 1.235 mmol/L.

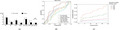

## 1 INTRODUCTION

It is estimated that the number of people with cardiovascular disease (CVD) in China is now about 330 million. In 2018, death of CVD accounted for the leading cause of total death among urban and rural residents in China, with 46.66% in rural areas and 43.81% in urban areas.^1^ The main pathological basis of CVD is atherosclerosis (AS).^2^ Abnormalities in the metabolism of low‐density lipoprotein (LDL) and total cholesterol (TC) are the most important risk factors for AS, contributing to the formation of atherosclerotic plaques and ultimately leading to cardiovascular and cerebrovascular events.^3^

sdLDL is associated with AS, and an increased level of sdLDL has a certain predictive value for cardiovascular and cerebrovascular events.^4^, ^5^ Firstly, sdLDL is small and easy to get through the vascular wall, becoming an effective cholesterol source for the formation of atherosclerotic plaque.^6^ Secondly, the low sialic acid content of sdLDL readily binds to anionic proteoglycans in the vessel wall, increasing the retention time of sdLDL in the subendothelial space of arterial vessels.^3^ Moreover, sdLDL has few polar molecules on its surface and is susceptible to various chemical modifications such as glycosylation,^7^ which is subsequently phagocytosed by macrophages and accelerates the formation of foam cells.^8^ In addition, sdLDL contains a few antioxidant vitamins^9^ and is more likely to become oxidized LDL (OX‐LDL) than larger forms of lipoproteins, generating oxidation‐specific epitopes, inducing immune responses and inflammation, and converging chemotactic monocytes to vascular endothelial cells, while being able to generate antigen–antibody complexes to induce the formation of more foam cells to accumulate within the arterial wall and form the lipid core portion of atherosclerotic plaques.^10^, ^11^ OX‐LDL is cytotoxic, damaging the vascular endothelium, throwing off homeostasis, and accelerating the formation of atherosclerotic plaques.^2^ The increase of triglyceride levels in plasma can promote the transformation of LDL from lbLDL to sdLDL^12^ and increase the risk of AS.^13^

Studies have shown that sdLDL is closely related to the risk of CVD, and the National Cholesterol Education Program (NCEPIII) lists sdLDL as one of the risk factors for CVD.^14^ To prevent and control cardiovascular and cerebrovascular events in high‐risk stroke patients at an early stage, this study aimed to investigate the value of sdLDL levels in high‐risk stroke patients for early prediction of cardiovascular and cerebrovascular events, provide a scientific basis for risk stratification management and early prevention in high‐risk stroke patients, and strive to lighten the heavy burden caused by CVD.

## 2 METHODS

### 2.1 Study subjects

**FIGURE 1:**
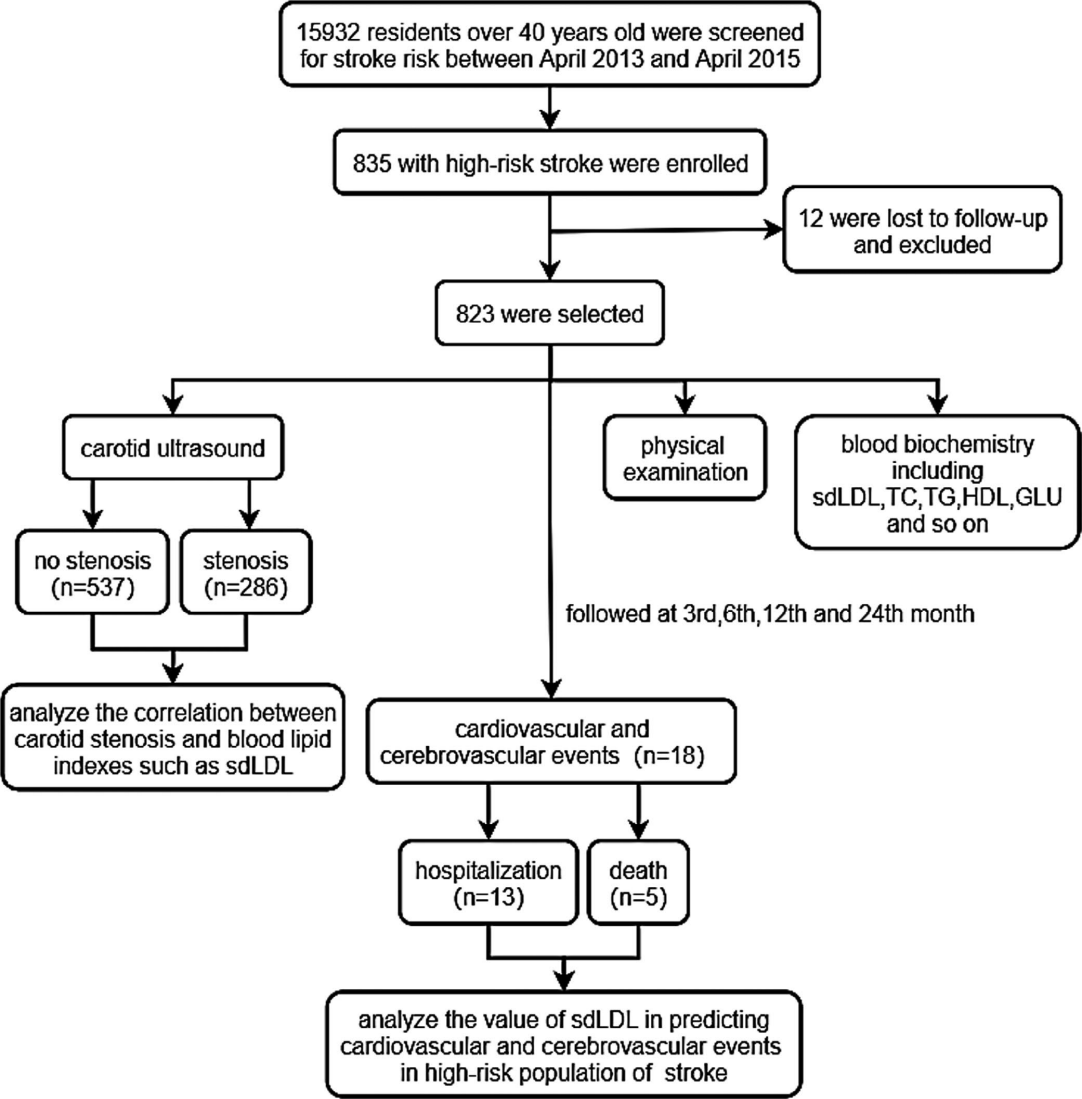
Flow chart of subjects selection. There were 835 cases enrolled from 15933 individuals who were screened for stroke risk, 12 cases lost to follow‐up, and 823 cases finally met the criteria, including 473 female and 350 male cases. A total of 286 cases with carotid artery stenosis, and 18 cases experienced cardiovascular and cerebrovascular events

This was a prospective study. High‐risk stroke patients were selected in April 2013 from the information of 15933 residents older than 40 years old in Chaohui Street, Xiacheng District, Hangzhou, Zhejiang Province. We enrolled 835 subjects, 12 cases were lost in the two‐year follow‐up, and 823 cases finally met the criteria, including 473 female and 350 male subjects. During the two‐year follow‐up, there were 18 cardiovascular and cerebrovascular events, including 13 hospitalizations and 5 deaths **(r**esearch flow chart is shown in Figure 1). Stop taking lipid‐lowering drugs for 4 weeks before enrollment.

Inclusion criteria: 8 risk factors were assessed according to the risk of stroke for people at high risk of stroke, those with 3 or more risk factors were considered high risk. Stroke‐risk screening assessment criteria: For people aged 40 years or older, stroke risk screening assessment was performed based on the following 8 risk factors (a) history of hypertension (systolic blood pressure ≥140/90 mmHg) or taking antihypertensive drugs; (b) heart disease such as heart valve disease and/or atrial fibrillation; (c) dyslipidemia; (d) diabetes mellitus; (e) obesity or significant overweight (body mass index ≥26 kg/m^2^); (f) lack of physical activity; (g) smoking; (h) family history of stroke.

Exclusion criteria: living or working outside the study area for more than six months; having severe liver or kidney disease or malignancy, psychiatric disease, or systemic immune disease; having incomplete information or missing visits during the 2‐year follow‐up cycle.

### 2.2 China stroke prevention project committee (CSPPC) stroke program

Stroke is of high incidence, disability, and mortality, which is the first cause of burden for disease in China. To reduce the burden of stroke, the former Chinese Ministry of Health established the CSPPC in April 2011. CSPPC formulates policies, issues clinical guidelines, and organizes community hospitals to carry out stroke risk factor screening and risk assessment for permanent residents over 40 years old in high‐incidence areas and to conduct health education and regular physical examinations for selected low‐risk populations, intervention guidance for the middle‐risk population based on individual characteristics, further inspections for the high‐risk population, and comprehensive intervention. During regular follow‐up of the middle‐risk and high‐risk groups, patients identified to have cervical vascular disease or suspected stroke will be referred to the hospital for further diagnosis and treatment.^15^ Our Stroke Prevention and Control Center is one of the members of CSPPC, and this project is jointly conducted with our Stroke Prevention and Control Center according to the task under CSPPC.

### 2.3 Carotid ultrasound and other information

Ultrasound examination of the neck was performed using a diagnostic ultrasound machine (Siemens S2000, Germany). The examined vessels mainly included bilateral common carotid arteries (CCA), carotid sinus (ICS), internal carotid artery (ICA), subclavian artery (SA), and vertebral artery (VA) to observe the smoothness of the vessel wall, the presence of plaque and carotid artery stenosis. Carotid artery stenosis was classified as (i) mild stenosis with 1%–49% reduction in internal diameter, with ultrasound images showing localized plaque and no significant changes in blood flow; (ii) moderate stenosis with 50%–69% reduction in internal diameter, accelerated blood flow at the plaque stenosis, and formation of pathological vortex distal to the stenosis; (iii) severe stenosis with 70%–99% reduction in internal diameter, aggravated plaque, further accelerated blood flow at the plaque stenosis, and distal A mixed‐signal of pathological vortex and turbulence was formed.

Demographic parameters such as age, gender, waist circumference, BMI, systolic blood pressure, diastolic blood pressure, a medical history of all subjects such as a history of heart disease, history of diabetes, history of hypertension, history of dyslipidemia, history of smoking, family history of hypertension, and family history of diabetes such as stroke data, family history of coronary heart disease, family history of stroke, carotid ultrasound findings, cardiovascular and cerebrovascular events were obtained from the China Stroke Data Center database.

Follow‐up: All enrollees were followed up by standardized trained community physicians, either as an outpatient or by telephone at 3rd, 6th, 12th, and 24th months during the two years from April 2013 to April 2015. The main content of the follow‐up visits was whether the enrollees had a cardiovascular and cerebrovascular event. No laboratory tests were performed during the follow‐up. Cardiovascular and cerebrovascular events are defined as death due to stroke or heart disease, or admission due to coronary heart disease, nonfatal myocardial infarction, stroke, and transient ischemic attack (TIA). After verification and confirmation, the data were entered into the database of the China stroke data center.

### 2.4 Determination of sdLDL and other biochemical indexes

The data were the laboratory tests performed once before the follow‐up of the included patients. For all subjects, fasting serum samples were collected within 24 h, 3 ml of cubital venous blood was drawn into a separating gel‐accelerating vacuum blood collection tube and left for 15 min after the plasma was precipitated. The serum was separated by centrifugation at a relative centrifugal force of 560 *g* for 15 min. One part was sent to the laboratory biochemistry room to test sdLDL, total cholesterol (TC), LDL, high‐density lipoprotein (HDL), glucose (GLU), triglycerides (TG), uric acid (UA), free fatty acids (FFA), Lp‐PLA2, high‐sensitivity C‐reactive protein (hs‐CRP) and lipoprotein an LP (a) on the same day. The other portion was immediately stored at −80°C.

sdLDL was tested by a peroxidase assay(Beijing Jiuqiang Biotechnology Co., Ltd., OLYMPUS). The reference laboratory sdLDL value is less than 1.362 mmol/L, and we considered high levels of those greater than 1.362 mmol/L. TC and TG were tested by an enzymatic method (GPO‐PAP method), GLU was tested by a hexokinase method, UA was tested by a uric acid enzymatic method (Beckman‐Coulter Laboratory Systems Ltd., OLYMPUS). LDL was tested by surfactant removal method, HDL was tested by selective inhibition method, FFA was tested by an enzymatic method (ACS‐ACOD method) (Sekisui Medical Co., Ltd., OLYMPUS). Hs‐CRP was tested by immunoturbidimetry, Lp(a) was tested by latex immunoturbidimetry (Beijing Lidman Biochemical Co., Ltd, OLYMPUS). Lp‐PLA2 was tested by rate method (Chongqing Zhongyuan Biotechnology Co., Ltd, OLYMPUS).

### 2.5 Cardiovascular and cerebrovascular event

Death due to stroke or heart disease, or coronary heart disease, nonfatal myocardial infarction, stroke, and transient ischemic attack (TIA).

### 2.6 Statistical analysis

Statistical analysis was performed using SPSS (version 20.0), and graphs were created using GraphPad Prism (version 9). Categorical variables were expressed as numbers (percentages, %), and continuous variables were expressed as mean ± standard deviation. Comparisons between groups were analyzed by the chi‐square test. Two groups were compared by *t* test, and multiple groups were compared by single‐factor analysis of variance. Data for non‐normally distributed measurements as median (interquartile range) and comparisons between groups were made by rank‐sum test. Correlation analysis was performed using Spearman. Survival analysis was performed using the proportional hazards regression model (Cox regression analysis) method, and *p* < 0.05 was considered to be statistically significant.

## 3 RESULTS

### 3.1 Relationship between sdLDL and carotid artery stenosis

A total of 823 subjects were enrolled in this study. According to the neck ultrasound findings, there were 537 cases in the no stenosis group and 286 cases in the stenosis group. The age, systolic blood pressure, proportion of males, and proportion of diabetes history were higher in the stenosis group than in the no stenosis group, and the difference between the two groups was statistically significant (*p *< 0.05) (Table 1). The sdLDL in the stenosis group was higher than that in the no stenosis group, and the difference was not statistically significant (*p* = 0.317).

**TABLE 1:** Baseline characteristics of subjects in the no stenosis and stenosis groups

	Degree of carotid artery stenosis		*p* Value
	No stenosis (*n* = 537)	Stenosis (*n* = 286)	
Age (year)	66.46 ± 8.38	71.1 ± 7.92	0.000
BMI (kg/㎡)	24.84 (22.82﹣27.01)	24.71 (22.64﹣26.77)	0.278
SBP (mm Hg)	135 (128﹣146)	143 (133﹣154)	0.000
DBP (mm Hg)	81.67 ± 10.28	82.03 ± 11.16	0.646
Male, *n* (%)	202 (37.6%)	148 (51.7%)	0.000
Heart Disease, *n* (%)	111 (20.7%)	65 (22.7%)	0.493
Diabetes, *n* (%)	207 (38.5%)	136 (47.6%)	0.013
Hypertension, *n* (%)	485 (90.3%)	265 (92.7%)	0.261
Dyslipidemia, *n* (%)	332 (61.8%)	173 (60.5%)	0.708
Smoking, n (%)	80 (14.9%)	41 (14.3%)	0.828
History of stroke, n (%)	138 (25.7%)	78 (27.3%)	0.625
TC (mmol/L)	5.43 ± 1.17	5.36 ± 1.11	0.413
TG (mmol/L)	1.70 (1.22﹣2.41)	1.76 (1.31﹣2.53)	0.565
LDL (mmol/L)	3.09 ± 0.89	3.13 ± 0.88	0.504
HDL (mmol/L)	1.22 (1.05﹣1.49)	1.20 (1.04﹣1.42)	0.160
non‐HDL (mmol/L)	4.13 ± 1.14	4.11 ± 1.04	0.743
GLU (mmol/L)	5.57 (5.00﹣6.66)	5.86 (5.09﹣7.27)	0.020
HOMAIR	2.49 (1.69﹣3.65)	2.38 (1.69﹣3.93)	0.894
HOMAIS	0.40 (0.27﹣0.60)	0.43 (0.25﹣0.59)	0.886
UA (μmol/L)	337 (279﹣402)	354 (295﹣411)	0.026
FFA (μmol/L)	584 (449﹣785)	609 (431﹣804)	0.820
hs‐CRP (mg/L)	0.73 (0.34﹣1.88)	0.73 (0.34﹣1.95)	0.518
LP(a) (g/dl)	1.174 (0.583﹣2.21)	1.228 (0.616﹣2.476)	0.180
sdLDL (mmol/L)	0.917 (0.651﹣1.228)	0.947 (0.676﹣1.248)	0.317
Lp‐PLA2 (IU/L)	554.04 ± 127.32	577.27 ± 135.16	0.015

Note

Categorical variables were expressed as numbers and proportions (%), continuous variables as mean ± standard deviation. Data for non‐normally distributed measurements were expressed as median (interquartile range).

HOMA‐IR = fasting blood glucose × Fasting insulin/22.5; Homa‐is = 1/(fasting blood glucose) × Fasting insulin).

Abbreviations: BMI, body mass index; DBP, diastolic blood pressure; FFA, free fatty acid; GLU, glucose; HDL, high‐density lipoprotein; LDL, low‐density lipoprotein; LP(a), Lipoproteins(a); LP‐PLA2, lipoprotein‐associated phospholipase A2; non‐HDL, non–high‐density lipoprotein cholesterol; SBP, systolic blood pressure; sdLDL, small dense low‐density lipoprotein; TC, total cholesterol; TC,total cholesterol; TG, triglycerides; UA, uric acid.

**FIGURE 2:**
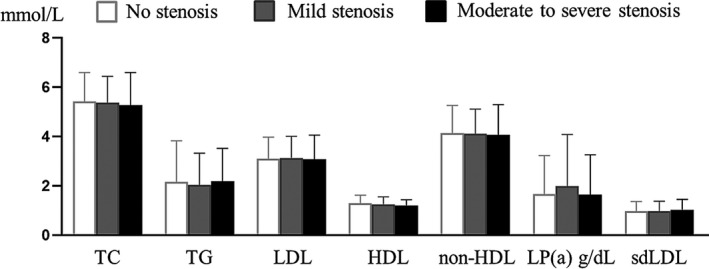
Levels of seven indicators with different degrees of stenosis in the three groups. The differences were not statistically significant (*p *> 0.05)

**FIGURE 3:**
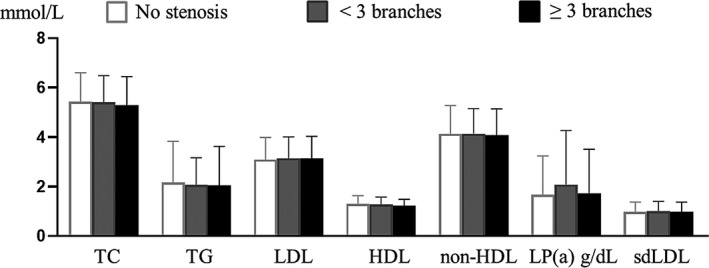
Levels of seven indicators with different narrow involvement range in the three groups. The differences were not statistically significant (*p *> 0.05)

The Spearman correlation analysis showed that the lipid indicators including TC, TG, LDL, non‐HDL, and Lp‐PLA2 were positively correlated with sdLDL (*r* > 0, *p *< 0.05), and Lp‐PLA2 was highly correlated with sdLDL in these high‐risk stroke populations(*r* = 0.555, *p *< 0.001) (Table 2). According to the degree of carotid artery stenosis, the three groups were divided into the no stenosis group (*n* = 537), mild stenosis group (*n* = 254), and moderate to severe stenosis group (*n* = 32). sdLDL increased with the severity of carotid artery stenosis, but the differences of 7 indicators including sdLDL among the three groups were not statistically significant (*p *> 0.05) (Figure 2). According to the number of carotid artery stenosis, the three groups were divided into the group with no stenosis, the group with less than 3 stenoses (involving 1–2 carotid arteries) and the group with more than or equal to 3 stenoses (involving 4–6 carotid arteries), the differences of 7 indicators including sdLDL among the three groups were not statistically significant (*p *> 0.05) (Figure 3).

**TABLE 2:** Correlation analysis between sdLDL and other indicators

	sdLDL	
	*r*	*p*
TC	0.734	0.000
TG	0.633	0.000
LDL	0.687	0.000
HDL	−0.103	0.003
non‐HDL	0.814	0.000
LP(a)	−0.024	0.493
Lp‐PLA2	0.555	0.000

This study also analyzed the relationship between the grouping of sdLDL quartiles and the incidence of carotid stenosis. The incidence of carotid stenosis increased with increasing sdLDL levels but without statistical significance (*p *> 0.05). In the grouping with or without stenosis and the grouping of stenosis severity, the incidence of the fourth quartile in the mild stenosis group and the fourth quartile in the moderate to severe stenosis group were the highest among the four groups, and the differences were not statistically significant (*p *> 0.05) (Table 3)

**TABLE 3:** Relationship between carotid artery stenosis and sdLDL levels

	Quartiles of sdLDL				*p*
	Q1	Q2	Q3	Q4	
Carotid artery stenosis					
No stenosis	140 (68%)	135 (65.9%)	132 (64.1%)	130 (63.1%)	0.745
Stenosis	66 (32%)	70 (34.1%)	74 (35.9%)	76 (36.9%)	
Degree of carotid artery stenosis					
No stenosis	140 (68%)	135 (65.9%)	132 (64.1%)	130 (63.1%)	0.908
Mild stenosis	59 (28.6%)	64 (31.2%)	65 (31.6%)	66 (32.0%)	
Moderate to severe stenosis	7 (3.4%)	6 (2.9%)	9 (4.4%)	10 (4.9%)	
Carotid artery stenosis range					
No stenosis	140 (68%)	135 (65.9%)	132 (64.1%)	130 (63.1%)	0.574
<3 branches	40 (19.4%)	39 (19%)	52 (25.2%)	49 (23.8%)	
≥3 branches	26 (12.6%)	31 (15.1%)	22 (10.7%)	27 (13.1%)	

Note

First quartile (Q1): sdLDL ≤0.659 mmol/L, *n* = 206; Second quartile (Q2): 0.659 mmol/L< sdLDL <0.926 mmol/L, *n* = 205; Third quartile (Q3): 0.926 mmol/L ≤ sdLDL <1.235 mmol/L, *n* = 206; Fourth quartile (Q4): sdLDL ≥1.235 mmol/L, *n* = 206.

### 3.2 sdLDL predicts cardiovascular and cerebrovascular events in people at high risk of stroke

**FIGURE 4:**
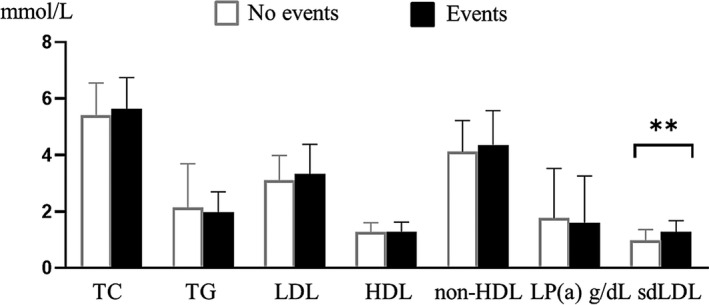
Difference of 7 indicators between the group without cardiovascular and cerebrovascular events and the group with cardiovascular and cerebrovascular events, **p < 0.01

**FIGURE 5:**
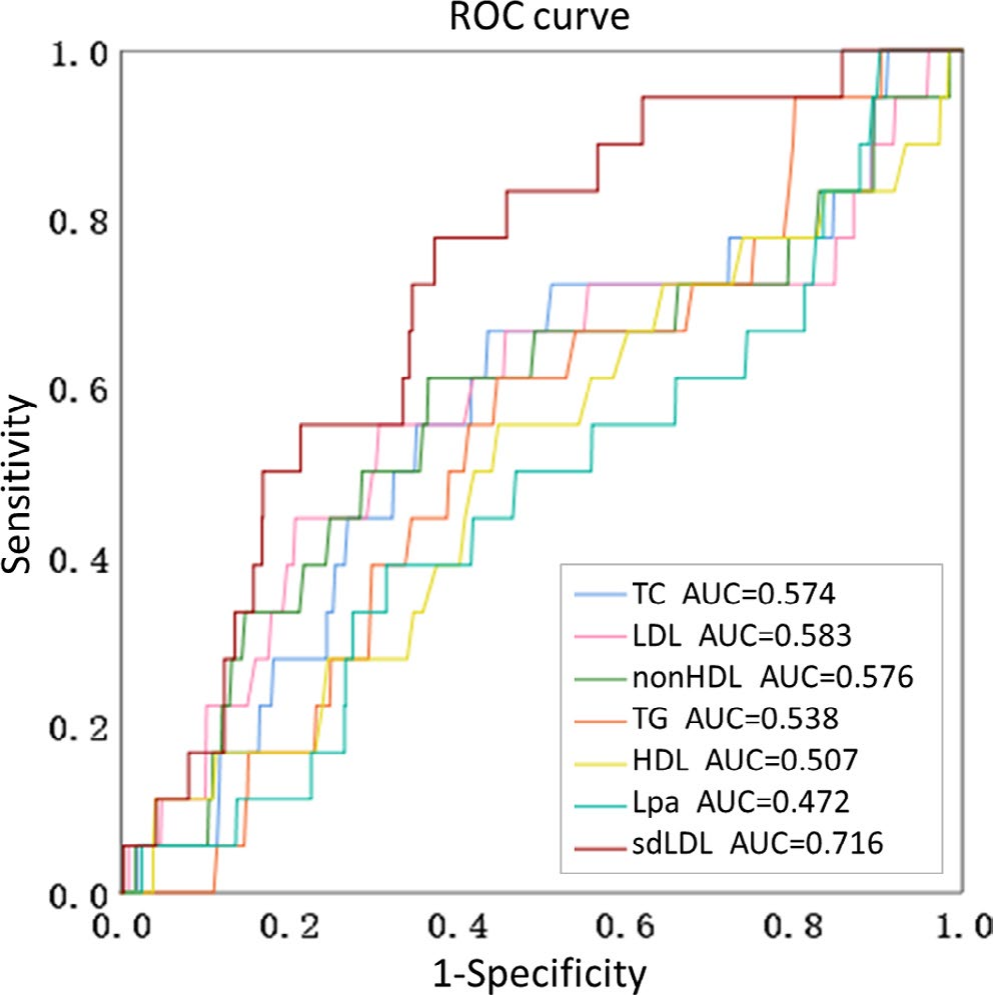
Predictive values of serum lipids for cardiovascular and cerebrovascular events. The ROC curves and AUCs regarding risk are depicted in Figure 5. AUCs: 0.716 for sdLDL, 0.574 for TC, 0.583 for LDL, 0.576 for nonHDL, 0.538 for TG, 0.507 for HDL, 0.472 for Lpa

During the 2‐year follow‐up of the 835 high‐risk stroke patients enrolled, a total of 18 cardiovascular and cerebrovascular events occurred, 11 of which were strokes. The differences in the incidence of cardiovascular and cerebrovascular events in the carotid stenosis group and the degree of stenosis involvement group were not statistically significant (*p *> 0.05) (Table 4), which were not predictive of cardiovascular and cerebrovascular events in high‐risk stroke patients. However, sdLDL predicted the occurrence of cardiovascular and cerebrovascular events in high‐risk stroke patients. sdLDL levels were significantly higher in the group with cardiovascular and cerebrovascular events than in the group without events(*p* = 0.002), which was statistically different (Figure 4). sdLDL levels of stroke patients are higher than non‐stroke patients1.383(1.069–1.551) versus 0.924 (0.658–1.231) mmol/L, *p* = 0.005). According to the ROC curve, an optimal value of 1.069 mmol/L produced a sensitivity of 77.8% and a specificity of 62.7%, with AUC (95% CI) of 0.716 (0.612–0.819), which shows a significantly greater discriminatory ability as compared with other serum lipids. This indicated that serum sdLDL level had a better risk prediction for cardiovascular and cerebrovascular events than other lipid parameters, including TC, TG, LDL, HDL, nonHDL, and LP (a) (Figure 5).

**TABLE 4:** Relationship between carotid artery stenosis and the incidence of cardiovascular and cerebrovascular events in high‐risk stroke patients

	Cardiovascular and cerebrovascular events		*p*
	No events	Events	
Carotid artery stenosis			
No stenosis	528 (98.3%)	9 (1.7%)	0.170
Stenosis	277 (96.9%)	9 (3.1%)	
Degree of carotid artery stenosis			
No stenosis	528 (98.3%)	9 (1.7%)	0.156
Mild stenosis	247 (97.2%)	2 (6.2%)	
Moderate to severe stenosis	30 (93.8%)	9 (1.7%)	
Carotid artery stenosis range			
No stenosis	528 (98.3%)	9 (1.7%)	0.149
<3 branches	176 (97.8%)	4 (2.2%)	
≥3 branches	101 (95.3%)	5 (4.7%)	

**FIGURE 6:**
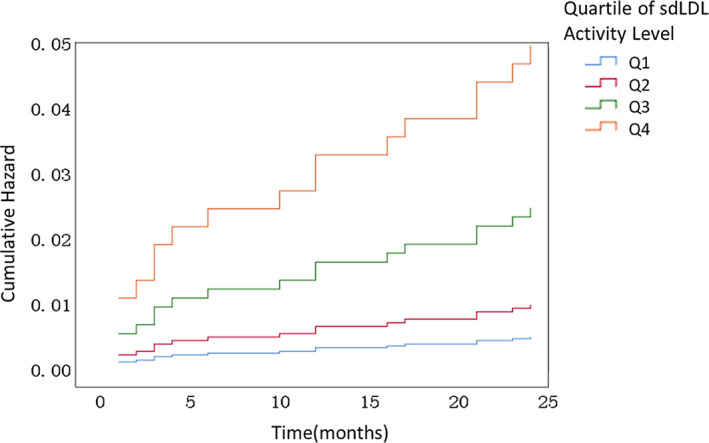
sdLDL quartile grouping hazard function diagram. Q1: sdLDL ≤0.659 mmol/L; Q2: 0.659 mmol/L< sdLDL <0.926 mmol/L; Q3: 0.926 mmol/L≤ sdLDL <1.235 mmol/L; Q4: sdLDL ≥1.235 mmol/L

In the subsequent analysis, using Cox regression to analyze the forward LR method, the occurrence of cardiovascular and cerebrovascular events was used as the value indicating that the event had occurred, the follow‐up time as a time variable, the sdLDL quartiles as a categorical covariate, and the first quartile of sdLDL quartiles as a reference group. The results suggested that sdLDL was a risk factor for the occurrence of cardiovascular and cerebrovascular events using the Cox regression forward LR method (*p* = 0.037). In the group of cardiovascular and cerebrovascular events, the incidence of cardiovascular and cerebrovascular events increased with the increase of the sdLDL quartile (*p* = 0.015) (Table 5). Compared to the first quartile, the risk of cardiovascular and cerebrovascular events in the fourth quartile of sdLDL was 10.136‐fold higher than the first quartile (HR = 10.14, 95% CI: 1.30–79.18, *p* = 0.027) (Table 6) (Figure 6).

**TABLE 5:** Relationship between the incidences of cardiovascular and cerebrovascular events after grouping sdLDL quartiles

	Quartiles of sdLDL				*p*
	Q1	Q2	Q3	Q4	
No events	205 (99.5%)	203 (99%)	201 (97.6%)	196 (95.1%)	0.015
Events	1 (0.5%)	2 (1%)	5 (2.4%)	10 (4.9%)	

Note

The differences between the events and no evens group of sdLDL with varying level. First quartile (Q1): sdLDL ≤0.659 mmol/L, *n* = 206; Second quartile (Q2): 0.659 mmol/L< sdLDL <0.926 mmol/L, *n* = 205; Third quartile (Q3): 0.926 mmol/L ≤ sdLDL <1.235 mmol/L, *n* = 206; Fourth quartile (Q4): sdLDL ≥1.235 mmol/L, *n* = 206.

**TABLE 6:** Cox regression analysis of sdLDL quartile grouping and cardiovascular and cerebrovascular events

	B‐value	SE	Wals	OR value	95% C.I.		*p*
					Upper bound	Lower bound	
Q1			8.48				0.037
Q2	0.70	1.23	0.33	2.01	0.18	22.20	0.568
Q3	1.62	1.10	2.19	5.05	0.59	43.24	0.139
Q4	2.32	1.05	4.88	10.14	1.30	79.18	0.027

Abbreviation: CI, confidence interval.

## 4 DISCUSSION

Cardiovascular and cerebrovascular events continue to be one of the leading causes of death and disability in the world.^16^ It is significant to know how to intervene scientifically and reduce the occurrence of cardiovascular and cerebrovascular events. Various biomarkers have been investigated to predict cardiovascular and cerebrovascular events in high‐risk stroke populations. For example, our team has published in this journal to predict cardiovascular and cerebrovascular events using Lp‐PLA2, which reflects the stability of atherosclerotic plaques.^17^ Inflammatory biomarkers, such as CRP, IL‐6, and TNF‐a, are often associated with pathogenic infections; CRP is significantly elevated in bacterial infectious diseases and can identify bacterial and viral infections; IL‐6 and TNF‐a are significantly elevated in cytokine storms during the acute phase of viral infectious diseases. Although there is a correlation between atherosclerotic plaque stability and low‐grade inflammation localized to the plaque, the sensitivity and specificity of using these indicators to predict cardiovascular and cerebrovascular events in a high‐risk stroke population are not high.^18^, ^19^, ^20^ Serum lipids are the main indicators for monitoring stroke risk, but conventional serum lipid indicators do not make much sense. LDL cholesterol is the main cholesterol causing atherosclerosis and is also the target of cholesterol‐lowering drugs, while small and dense LDL cholesterol is the main part of LDL cholesterol causing atherosclerosis. Compared with other serum lipid indicators, sdLDL has higher sensitivity to AS and reliable predictive value for cardiovascular and cerebrovascular events.^21^ Therefore, this study investigated the relationship between sdLDL and cardiovascular and cerebrovascular events, and the results showed that the sdLDL level in the cardiovascular event group was significantly higher than that in the no event group. sdLDL level was positively correlated with the incidence of cardiovascular and cerebrovascular events and increased with the severity of cardiovascular and cerebrovascular events, indicating that sdLDL level could predict the occurrence of cardiovascular and cerebrovascular events. In the ROC curve analysis, the AUC of sdLDL was higher than other lipid indices, suggesting its higher predictive value for cardiovascular and cerebrovascular event risk. In Cox regression analysis, the results showed that sdLDL was a risk factor for cardiovascular and cerebrovascular events in high‐risk stroke patients (*p* = 0.037), and the risk of cardiovascular and cerebrovascular events increased with increasing sdLDL quartiles, indicating that sdLDL levels have predictive value for cardiovascular and cerebrovascular events in high‐risk stroke patients, providing a scientific basis for risk stratification management and early prevention in high‐risk stroke patients.

sdLDL is considered a risk factor for cardiovascular disease in many diseases. Zafar et al.^22^ confirmed that sdLDL has a stronger AS potential and accelerates atherosclerotic plaque formation due to smaller particles, easier oxidation, a lower affinity for LDL receptors, and easier access to the arterial wall, explaining the relationship between sdLDL and cardiovascular and cerebrovascular risk. This is consistent with our findings that sdLDL levels were significantly higher in the cardiovascular event group than in the no cardiovascular event group. Our preliminary findings suggest the value of Lp‐PLA2 for clinical applications in predicting the occurrence of cerebrovascular events and evaluating the degree of carotid artery stenosis.^17^ Lp‐PLA2 plays a key role in the aggregation and activation of leukocytes and platelets, proliferation and migration of vascular smooth muscle cells, endothelial dysfunction, expression of adhesion molecules and cytokines, and formation of plaque necrosis core processes. The formation of Lp‐PLA2 downregulates endothelial nitric oxide synthesis and release, enhances oxidative stress response, and promotes endothelial cell apoptosis. sdLDL stays in the subendothelial space of arterial vessels for a long time, which is susceptible to be chemically modified, induces immune response and inflammation, promotes the formation of foam cells, and accelerates the development and progression of atherosclerotic plaques. In the correlation analysis of Lp‐PLA2 with other lipoproteins, sdLDL showed the strongest correlation with Lp‐PLA2 (*r* = 0.555). Lp‐PLA2 particles mainly bind to small and dense LDL particles, and in atherosclerotic plaques, Lp‐PLA2 hydrolyzes and oxidizes modified phospholipids in LDL particles to produce lysophosphatidylcholine (lyso‐PC) and oxidized unsaturated fatty acids, leading to atherosclerotic plaque instability and further affecting cardiovascular and cerebrovascular events. This may account for the higher sdLDL in the carotid stenosis group than in the no stenosis group of the results of this study, and the tendency for sdLDL levels to increase with increasing severity of carotid stenosis, but the difference was not statistically significant.

Although stenosis caused by carotid plaque is related to stroke, the degree of stenosis guided intracranial vascular interventional therapy has been controversial. In 2006, a GESICA study showed that whether patients with intracranial artery stenosis complicated with a hemodynamic disorder is the key factor of stroke, and the automatic regulation of cerebral blood flows is the main way of brain tissue self‐protection. Therefore, the risk of stroke caused by carotid stenosis is also related to the stability of plaque and the automatic regulation of cerebral blood flow. This may help explain why carotid stenosis cannot predict cerebrovascular events in this study.

Chen et al.^23^ found a statistically significant difference in sdLDL in the ischemic stroke group compared with normal controls (*p* = 0.001). Chen et al.^24^ found that sdLDL‐C was higher in the cerebral infarction group than in the cerebral hemorrhage group in a study of 652 stroke patients (*p *< 0.05), and the specificity of sdLDL‐C was 90.0%, which can be used as a risk assessment for cerebrovascular disease and has certain diagnostic value. Tu et al.^25^ reported that adiponectin was associated with a high risk of major adverse cardiovascular and cerebrovascular events and mortality. The subjects of this study were slightly different from the above‐mentioned studies. 823 high‐risk stroke patients were enrolled and followed up for 2 years, sdLDL was significantly higher in the group with cardiovascular and cerebrovascular events than in the group without cardiovascular and cerebrovascular events (1.315 (1.045–1.455) versus 0.920 (0.658–1.230) mmol/L, *p* = 0.002). According to the ROC curve, sdLDL show a higher sensitivity to cardiovascular and cerebrovascular events as compared with adiponectin. Subdividing the occurrence of cardiovascular and cerebrovascular events group into hospitalization and death groups revealed that the sdLDL level of death group was the highest, which was 1.351 (1.111–1.424) mmol/L, and sdLDL levels increased with the increasing severity of cardiovascular and cerebrovascular events. In the group with cardiovascular and cerebrovascular events, compared with the first quantile of sdLDL, the risk of cardiovascular and cerebrovascular events in the fourth quantile was 10.136 times higher than that in the first quantile (OR = 10.136, 95% CI: 1.298–79.180, *p* = 0.027). The results suggest that sdLDL is a risk factor for cardiovascular and cerebrovascular events in patients with high‐risk stroke (*p* = 0.037), and the risk of cardiovascular and cerebrovascular events increases with quartiles. These findings confirm the value of the clinical application of sdLDL levels in predicting the occurrence of cardiovascular and cerebrovascular events in high‐risk stroke patients.

The limitations of this study are as follows. Firstly, the volume of specimens included was not large, the follow‐up period was only 2 years, and the number of cases of cardiovascular and cerebrovascular events that eventually occurred was relatively small. Secondly, the subjects included were high‐risk stroke patients among residents aged 40 years or older in Chaohui Street, Xiacheng District, Hangzhou, and the results are only representative of a small population. In the subsequence, we will increase the sample size and continue follow‐up to obtain more and more comprehensive data for further exploration and analyze the clinical value of sdLDL in predicting cardiovascular and cerebrovascular events.

In conclusion, sdLDL levels are positively correlated with the incidence of cardiovascular and cerebrovascular events, which can predict the occurrence of cardiovascular and cerebrovascular events and provide a scientific basis for risk stratification management and early prevention in people with high risk of stroke.

## CONFLICT OF INTEREST

The authors declare that they have no competing interests.

## Data Availability

The data that support the findings of this study are available from the corresponding author upon reasonable request.
